# Conserving the genetic diversity of condemned populations: Optimizing collections and translocation

**DOI:** 10.1111/eva.13192

**Published:** 2021-02-01

**Authors:** Jason G. Bragg, Jia‐Yee S. Yap, Trevor Wilson, Enhua Lee, Maurizio Rossetto

**Affiliations:** ^1^ Research Centre for Ecosystem Resilience Australian Institute of Botanical Science, The Royal Botanic Garden Sydney Sydney NSW Australia; ^2^ School of Biological Earth and Environmental Sciences University of New South Wales Sydney NSW Australia; ^3^ Queensland Alliance of Agriculture and Food Innovation University of Queensland Santa Lucia QLD Australia; ^4^ Biodiversity and Conservation Division Department of Planning, Industry and Environment Parramatta NSW Australia

**Keywords:** collections, conservation, genetic diversity, plant, translocation

## Abstract

We consider approaches for conserving genetic diversity from plant populations whose destruction is imminent. We do this using SNP genotype data from two endangered species, *Pimelea spicata* and *Eucalyptus* sp. Cattai. For both species, we genotyped plants from a ‘condemned’ population and designed ex situ collections, characterizing how the size and composition of the collection affected the genetic diversity preserved. Consistent with previous observations, populations where genetic diversity was optimized captured more alleles than populations of equal size chosen at random. This benefit of optimization was larger when the propagation population was small. That is, small numbers of individuals (e.g. 20) needed to be selected carefully to capture a comparable proportion of alleles to optimized populations, but larger random populations (e.g. >48) captured almost as many alleles as optimized populations. We then examined strategies for generating translocation populations based on the horticultural constraints presented by each species. In *P. spicata*, which is readily grown from cuttings, we designed translocation populations of different sizes, using different numbers of ramets from each member of propagation populations. We then performed simulations to predict the loss of alleles from these populations over 10 generations. Large translocation populations were predicted to maintain a greater proportion of source population alleles than smaller translocation populations, but this effect was saturated beyond 200 individuals. In *E*. sp. Cattai, we examined strategies to promote the diversity of progeny from a conservation planting scenario with 36 individuals. This included the optimization of the spatial arrangement of the planting and supplementing the diversity of the condemned population with individuals from additional sites. In sum, we studied approaches for designing genetically diverse translocations of condemned populations for two species that require contrasting methods of propagation, illustrating the application of approaches that were useful in different circumstances.

## 1 INTRODUCTION

Human activity impacts the habitat of a vast number of species, and many species are threatened with extinction as a consequence (IPBES, 2019). The drivers are manifold and include the transformation of land for agriculture and urban development. The disturbance of previously continuous habitat can reduce the sizes of natural populations, and leave them fragmented (Haddad et al., 2015). This exposes organisms to substantial genetic risks, including local inbreeding and associated loss of fitness (inbreeding depression), and compromised adaptive capacity (Templeton et al., 1990; Young et al., 1996).

**FIGURE 1:**
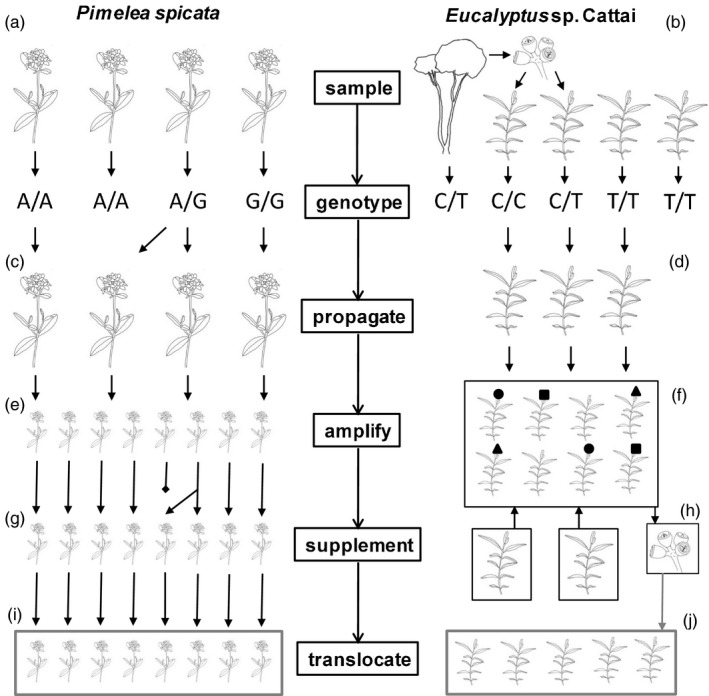
Workflows for preserving the genetic diversity in condemned populations. Steps are depicted for the design of ex situ collections and translocation populations for two exemplar species with different life‐history and horticultural properties. The workflow starts with the collection of a tissue sample from many individuals in the condemned population (a, b). This should include all individuals that could be used in the ex situ collection. For example, in the case of *Eucalyptus* sp. Cattai, which cannot be grown from cuttings, this involved collecting seeds from the condemned population, growing seedlings and sampling the seedlings in addition to the adult trees (b). The next step is to genotype all the samples and to use the genotypic information to design and propagate an optimized ex situ collection (c, d). From here, the subsequent steps depend very strongly on the horticultural properties of the species and the plan for its conservation. One possible path is to amplify the collection by taking cuttings (e) and to translocate these to another site, as depicted for *Pimelea spicata* (i). Where propagation from cuttings is not possible, such as for *E*. sp. Cattai, amplification might instead be performed by establishing a conservation planting (f) and harvesting progeny from it (e.g. a seed orchard) (h) for translocation (j). We describe an approach we used to optimize the spatial arrangement of individuals in a hypothetical planting, to separate genetically similar individuals (as illustrated using the same shapes; f). We have also developed approaches to supplement ex situ collections to maximize diversity (g, h). This can be useful if there is mortality in a collection or a translocated population (see Bragg et al., 2020) (g). Here we present an approach to supplement plant material from a single site (e.g. a condemned population) with individuals from additional sites (h). This can increase the genetic diversity of the planting, but potentially also brings the risk of swamping diversity that is being preserved

In some landscapes, particularly near urban areas, habitat disturbance is both extensive and ongoing. This means species that have experienced contractions in the extent of their habitat will be subject to further reductions, in addition to changes in other evolutionary processes (Rivkin et al., 2019). Sometimes the loss of a population of an already endangered species is unavoidable. We will refer to such populations – whose destruction is imminent – as ‘condemned’ and consider approaches for preserving the genetic diversity represented in them. The preservation of genetic diversity can be achieved in the form of ex situ germplasm collections or the establishment of new populations in natural areas (Frankham et al., 2017) (Figure 1). Ideally, these collections and translocations would aim to conserve as much genetic diversity as possible (Guerrant, 1996; Vallee et al., 2004), though constraints are imposed on ex situ collections and translocation populations by plant traits (e.g. generation time) and the resources available for propagation or storage.

The resource limitations on the propagation of threatened plant species are best illustrated by the scope of the problem. In Australia, 748 species of flora are listed as Endangered or Critically Endangered under the *Environment Protection and Biodiversity Conservation Act 1999* (hereafter, EPBC Act), and many of these have small populations. If we want to maximize the number of populations and species that can be preserved in ex situ collections and translocations, we need to understand how to undertake these actions as efficiently as possible in terms of time, space and materials. This emphasis on efficiency leads to a variety of trade‐offs in the design of translocation populations. For example, it is typically possible to conserve more genetic variation (e.g. more alleles) by maintaining a greater number of individuals in a collection but at the cost of using more resources or nursery space (e.g. Griffith & Husby, 2010, Hoban et al., 2020, 2018, Hoban & Schlarbaum, 2014, Marshall & Brown, 1975, Richards et al., 2007).

The properties of different plants heavily influence the ways in which translocation populations can be obtained. These properties include plant life histories and horticultural characteristics. Here it is useful to divide the process of making a translocation population into two phases. The first consists of taking material (cuttings or seeds) from the source population and then propagating it, for example in a nursery. This is similar to the maintenance of a germplasm core set for a crop, and the process can similarly be approached with the goal of maximizing the diversity and representativeness of the collection (e.g. Gouesnard et al., 2001, Hoban, 2019, Marshall & Brown, 1975, Reeves & Richards, 2017, Richards et al., 2007, Schlottfeldt et al., 2015, Schoen & Brown, 1993). The second is to establish a translocation population, ideally with a larger size than the propagation population (‘amplification’), using cuttings or seeds from the propagated material (Guerrant, 1996; Vallee et al., 2004; Weeks et al., 2011). The feasibility of amplification, and the approaches used, will differ greatly among plants, depending on their life‐history traits (e.g. generation time) and horticultural properties (e.g. whether they can be grown from cuttings). The ultimate goal is to establish a self‐sustaining population in a natural environment and to eventually obviate the need for the ex situ propagation population.

Here we examine strategies for designing translocation populations that aim to conserve genetic diversity from a condemned source population, using the framework of a broadly applicable workflow (Figure 1). The workflow aims to be efficient, in using a single round of genotyping to inform a sequence of management activities, including the selection of material from the condemned site for an ex situ collection, followed by the amplification of germplasm for use in a translocation. The implementation of these steps varies among species according to differences in species properties and specific planned translocation objectives. We apply the workflow to empirical data sets for two endangered species, *Pimelea spicata* and *Eucalyptus* sp. Cattai. In both cases, a ‘condemned’ population facing imminent destruction has been genotyped, and we design optimized propagation populations. We consider approaches for the generation of diverse translocation populations in light of different horticultural constraints, via the use of cuttings (*P. spicata*) and the design of a spatially optimized ex situ planting (*E*. sp. Cattai).

## 2 MATERIALS AND METHODS

### 2.1 Overview

We present and apply a workflow for assembling ex situ collections and designing translocation populations from these, with the aim of conserving the genetic diversity in condemned source populations (Figure 1). We briefly describe the study species, *P. spicata* and *Eucalyptus* sp. Cattai (Section 2.2), and outline the molecular methods we used to study genetic variation within them (Section 2.3). We then describe the way the workflow was applied, in light of translocation goals and constraints, horticultural properties and the genetic data. This included the design of propagation and translocation populations for *P. spicata* (Sections 2.4 and 2.5), and a propagation population for *E*. sp. Cattai (Section 2.6), as well as the examination of strategies to promote genetically diverse progeny from a conservation planting of *E*. sp. Cattai (Sections 2.7 and 2.8).

### 2.2 Study species and translocation goals

**FIGURE 2:**
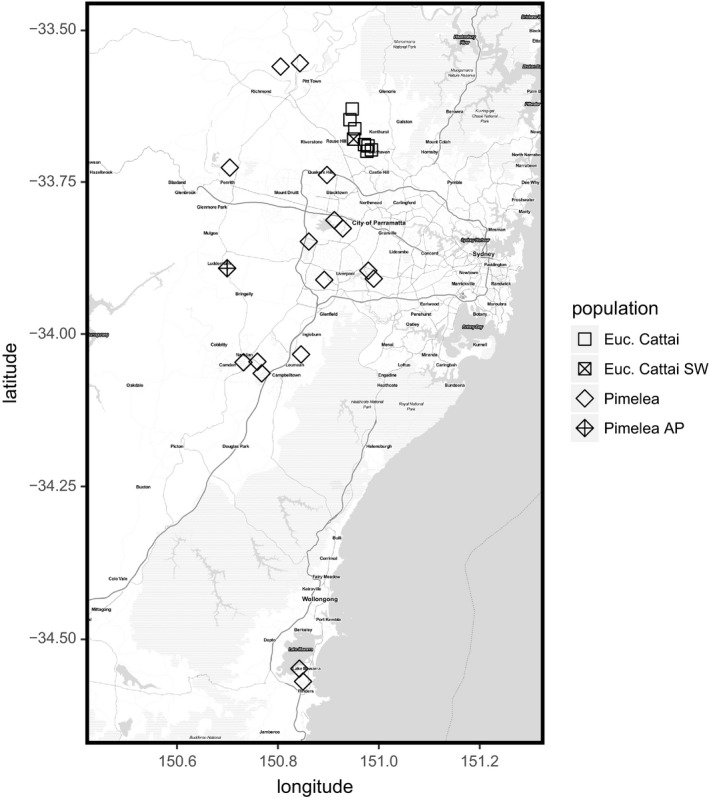
Map of sampled locations for *Pimelea spicata* and *Eucalyptus* sp. Cattai. The location the condemned population of each species (AP, airport and SW, saltwater, respectively) is indicated

*Pimelea spicata* R. Br. (Thymelaeaceae) is an endangered shrub, listed on both state (NSW *Biodiversity Conservation Act 2016*; hereafter, NSW BC Act) and federal (EPBC Act) legislation. It is perennial and can resprout from tap roots following disturbance. It is likely capable of self‐pollination, and although it grows prostrate branches, it is considered unlikely to be capable of vegetative reproduction in the wild. The range of *P. spicata* spans approximately 100 km (north to south) on the east coast of Australia (Figure 2). Across this range, it occurs in approximately 30 populations centred in two distinct regions, though it was probably more widely and more contiguously distributed prior to land clearing, especially around the Sydney metropolitan area. The habitat of one of the largest remaining populations of *P. spicata* will be destroyed during the construction of an airport – Western Sydney International (Nancy‐Bird Walton) Airport – in the suburb of Badgery's Creek, Sydney (hereafter, the ‘airport’ site). As part of the Biodiversity Offset Delivery Plan (BODP) for the airport site, and in accordance with the EPBC Act Offsets Policy, we set out to design a translocation population based on cuttings of *P. spicata* from the airport site, to compensate for the impacts of construction. The translocation was planned to occur in two phases. The first was the propagation of individuals from the condemned population (Figure 1c). We refer to this as the ‘propagation population’, and note that it represents an ex situ germplasm collection that will remain available after the initial translocation (e.g. for supplementation, or re‐establishment, in case of catastrophic losses). The second part of the plan was to establish a population (‘translocation’ population) at a new site using cuttings from individuals of the propagation population (Figure 1i). In each phase, our goal was to incorporate as much genetic variation as possible from the condemned population, while using as few plants as possible to reduce the resources needed for establishment and maintenance of plant material.

*Eucalyptus* sp. Cattai is a critically endangered tree under relevant state (NSW BC Act) and federal (EPBC Act) legislation. It often has a mallee growth habit. Little is known about the reproductive biology of the species, however eucalypts tend to hybridize inter‐specifically, and it is therefore plausible that *E*. sp. Cattai would hybridize with other *Eucalyptus* species in the region. *Eucalyptus* sp. Cattai has a severely restricted and fragmented distribution, occurring at approximately twelve sites in north‐western Sydney (Figure 2). One of the largest populations of *E*. sp. Cattai, at Saltwater Circle, Sydney (hereafter, the ‘saltwater’ site) has been cleared for development. A conservation project was developed under the *Saving Our Species* initiative by the Department of Planning, Industry and Environment (New South Wales Government), which identified translocation as a priority conservation action. Unlike *P. spicata*, *E*. sp. Cattai cannot be easily propagated from cuttings for translocation purposes, so we examined the design of ex situ populations using seedlings grown from seeds that were collected prior to clearing (Figure 1d). Our examination of translocation population design was performed in conjunction with a detailed ongoing study of the population genetics of *E*. sp. Cattai, as well as the taxonomy of the *Eucalyptus* clade (subgenus *Symphyomyrtus* section Latoangulatae) that includes it. This work supports the species status of *E*. sp. Cattai, which will be described in a future publication, and identified putative interspecific hybrids that were excluded from ex situ collection designs.

### 2.3 Genotyping and preliminary analyses

We collected leaf tissue samples from 282 *P. spicata* individuals from 16 sites across the range (Figures 1 and 2). In total, 120 of these samples were from the condemned population at the Western Sydney Airport (hereafter ‘airport’). This included 100 samples collected from the airport site, and 20 samples that had been collected previously and propagated at the Australian Botanic Gardens, Mt Annan (ABGMA). For *E*. sp. Cattai, we sampled 188 individuals for genotyping from eight sites. This included a total of 99 tissue samples from adult trees, and 89 from seedlings that were propagated from seeds collected at four sites (Figures 1 and 2). No *E*. sp. Cattai seedlings were observed at the collection sites. Sampling from the condemned ‘saltwater’ site consisted of 26 adults and 63 seedlings that were grown from seed at the ABGMA.

The handling of leaf samples followed the protocols outlined in Rossetto et al. (2019). We genotyped the leaf samples using DArTseq, which is a commercial genotyping service (Diversity Arrays Technology Pty Ltd). DArTseq is a reduced representation sequencing approach that involves performing a restriction digest of sample DNA, and then high throughput sequencing of the resulting digestion products (Sansaloni et al., 2010). In this sense, it is similar to RADseq or genotyping by sequencing (Andrews et al., 2016). We filtered the raw DArTseq genotype data using several quality criteria. Each DArT Single Nucleotide Polymorphism (SNP) locus is supplied with a ‘reproducibility’ score, which is an index of the precision of genotype calls between technical replicate samples. For all analyses, we used a reproducibility threshold of >0.96. For initial analyses, we also excluded SNP loci than were missing genotype calls for >20% of samples. We then performed a series of preliminary population genetic analyses (using R packages, R Core Team, 2016), including the estimation of population diversity measures (package diveRsity; Keenan et al., 2013), differentiation between populations and relatedness between individuals (package SNPRelate; Zheng et al., 2012).

For both species, it was possible we had sampled clonal individuals (ramets of a genet) or very close relatives. We set out to collect as much of the available diversity as possible, so when it was unclear if two stems were genetically identical (e.g. connected underground), we tended to sample both. However, we wanted to identify and remove the highly similar individuals prior to commencing the design of translocation populations. This is because genetically very similar individuals are likely to contribute few or no novel alleles, relative to each other. We used individual pairwise kinship, estimated using the PLINK method (Purcell et al., 2007, implemented in SNPRelate, Zheng et al., 2012), to assess the genetic similarity between samples. For genetically identical individuals, we expect kinship = 0.5, but estimated values tend to be <0.5 due to genotyping errors. This means it is not always trivial to identify an appropriate threshold that reliably distinguishes genetically identical individuals from pairs of individuals that are, for example, closely related and inbred. Therefore, we set threshold values of kinship for each species by examining the frequency distribution of observed kinship values (Appendix S1). The code used to filter genetic data and implement the analyses described above is available in a Dryad repository (doi:10.5061/dryad.5hqbzkh53).

### 2.4 An ex situ propagation population derived from cuttings: *Pimelea spicata*

For *P. spicata*, we first examined the establishment of a propagation population using cuttings of different subsets of the genotyped individuals (genets) from the condemned population (Figure 1c). We wanted to evaluate how the size of the propagation population (*N*_P_), and its composition (the specific selected plants) influenced the diversity that could be captured in it. We considered a range of propagation population sizes (*N*_P_ values of 16–64, in increments of 4) that reflected the scope of planned conservation activities and the desire to limit resource requirements. We then designed populations of these sizes in two ways. First, populations of each size (*N*_P_) were optimized for gene diversity. To do this, we used the simulated annealing optimization algorithm (Kirkpatrick et al., 1983) to choose a subset of the available plants that maximized the value of gene diversity (expected heterozygosity; Nei, 1973). Details for the implementation of simulated annealing are provided in Appendix S1. Second, we chose populations of each size (*N*_P_) at random from the genotyped individuals. This was repeated 100 times for each value of *N*_P_, and outcomes were averaged to inform the level of diversity that could be expected if individuals for the propagation population were selected at random. For each candidate population design, we began with the SNPs that were ‘common’ in the condemned population (minor allele frequency >2%) and calculated the proportion of these SNPs that were polymorphic in the candidate propagation population. We used this measure of the proportion of common alleles that were captured to compare candidate propagation populations of different sizes (different values of *N*_P_) and that were designed in different ways (optimized vs. random). For these analyses, we removed all loci with missing data. We did this to avoid making corrections for missing data when counting alleles in candidate propagation populations. Here we used a minor allele frequency of >2% as the threshold delimiting common SNPs. This represented a trade‐off between keeping data for as many SNPs as possible, while removing SNPs that might be especially subject to variation due to genotyping error (especially singletons).

### 2.5 A translocation population derived from cuttings: *Pimelea spicata*

The next step towards conserving the genetic diversity from the condemned *P. spicata* population was to design a translocation population using individuals derived from the propagation population. Here, we had the opportunity to use multiple cuttings (ramets) from each individual (genet) in the propagation population and to thus amplify the size of the translocation population (Figure 1e,i). We next wanted to understand how translocated populations of different sizes would perform in terms of the loss of alleles over generations. We therefore performed simulations in which different sized translocation populations were designed and simulated forward for 10 generations. Details of the simulations are provided in Appendix S1. We note that these simulations use a coarse description of life‐history and mating system, with some important parameters unknown (e.g. rates of selfing, variation in reproductive success among individuals). As such, it is possible the simulations provide biased predictions for the loss of variation by drift. Despite these limitations, we expect they provide a useful index for the relative rate of loss of diversity by different candidate translocation populations, assuming the unknown factors affect the candidate designs equally. Candidate translocation populations of different sizes (*N*_T_ = 64, 128, 192, 256, 320 individuals) were derived from optimized propagation populations of different sizes (*N*_P_ = 16, 32 and 64 individuals). For this analysis, whose goal was to explore the consequences of propagation and translocation population sizes on the conservation of alleles in the medium term, we used simple designs in which translocation populations had an equal number of ramets from each individual (genet) in the propagation population. In reality, different numbers of ramets of each individual could be used, either to further optimize genetic diversity or to allow for different levels of propagation success among individuals. In this exercise, for example, to construct a translocation population with *N*_T_ = 256 individuals from a propagation population of *N*_P_ = 32 individuals, we used eight ramets of each individual in the propagation population. Following a forward simulation of 10 generations, the number of SNP loci that remained polymorphic was calculated and expressed as a proportion of the total number of loci where the minor allele was common (>2%) in the condemned population.

### 2.6 An ex situ propagation population derived from seed: *Eucalyptus* sp. Cattai

For *E*. sp. Cattai, the conservation plan was to use a propagation population composed of seedlings that were harvested from the original site as seed and propagated ex situ (Figure 1b). We therefore wanted to know how much of the genetic diversity in the adult trees of the condemned population could be preserved within different sets of the available seedlings from the condemned population (Figure 1d). To address this, we used an optimization algorithm (simulated annealing, Kirkpatrick et al., 1983) to design candidate propagation populations of different sizes (*N*_P_ values of 20–52, in increments of 4) using the seedlings that had been collected from the saltwater site (excluding four putative interspecific hybrids). We then asked what proportion of common SNPs in the adult population (minor allele frequency >3%) were polymorphic in the candidate propagation populations that we designed. Similar to *P. spicata* (Section 2.4), we compared the proportion of alleles that were captured in these optimized propagation populations to the average proportion of alleles that were captured in populations of the same size that were chosen at random. For *E*. sp. Cattai, we present analyses of samples from adults and seedlings separately, in part because we wanted to know how well seeds represented an adult population. Here we used the minor allele frequency threshold of 3% because we wanted to remove singletons (frequency = 2.8%, with 18 diploid samples), which can result from a single genotyping error.

### 2.7 Supplementing a collection: *Eucalyptus* sp. Cattai

In *E*. sp. Cattai, where there is little prospect for using cuttings to amplify the material in propagation collections, we expect it will be particularly important to promote genetic diversity in progeny from an ex situ planting. Therefore, we examined additional approaches for bolstering this diversity, including the incorporation of a small number of seedlings from additional populations into the collection (Figure 1h). Specifically, we designed propagation populations (size *N*_P_ = 36) using a combination of the seedlings from saltwater, supplemented with seedlings from other sites (Gregson, *n* = 1; Clarke, *n* = 3; Logie, *n* = 4). We recognized that there might be a perceived trade‐off between the aims of designing a population that was as diverse as possible, and the possible impact of ‘swamping’ the diversity of the condemned population that we were trying to preserve. Therefore, we used multiobjective optimization to characterize any trade‐off that existed between maximizing gene diversity, and maximizing the representation of individuals from saltwater in the population. Details for the implementation of the multiobjective optimization are provided in Appendix S1.

### 2.8 The spatial arrangement of a conservation planting: *Eucalyptus* sp. Cattai

Finally, we examined an approach for optimizing the spatial arrangement of a propagation population, using the 36 seedlings chosen from Saltwater (Figure 1f). The aim was to reduce the frequency of inbreeding and increase the genetic diversity of the progeny from the planting by keeping genetically similar plants far apart and reducing the likelihood that they pollinate each other. To do this, we arranged the plants on a set of 36 points in space (layout shown in Figure 7a) in a way that minimized a function, *f*, describing the propensity for genetically similar plants to be near each other. The function, *f*, was follows:

$$f=\frac{1}{n_{\mathrm{pairs}}}\sum_{\mathrm{pairs}}\frac{1}{S_{i,j}}G_{i,j},$$

where subscripts *i* and *j* represent a pair of plants, *S_i_*_,_ *_j_* is the spatial distance between individuals *i* and *j*, *G_i_*_,_ *_j_* is a measure of the genetic similarity between individuals *i* and *j*, and *n*_pairs_ is the number of pairs of plants. In other words, if a given pair of plants, *i*, *j*, are genetically similar (large *G_i_*_,_ *_j_*), it will add a large value to *f* if they are close together (because 1/*S_i_*_,_ *_j_* will be large), and a small value if they are far apart (i.e. because 1/*S_i_*_,_ *_j_* will be small). This means *f* will be large if many genetically similar plants are close together, and smaller if genetically similar plants are far apart. We minimized *f* using a simulated annealing algorithm (see Appendix S1 for details). We note that the form and parameterization of *f* are flexible and could be informed by the reproductive biology of a given species, and the planting design. For *E*. sp. Cattai, the planting locations of individuals were arranged into ‘clumps,’ where a clump consisted of five individuals planted close together. This was because clumped planting arrangements have been shown to promote seed production in eucalypts (McCallum et al., 2019). However, the algorithm can be as easily run on a regular grid, if that is more appropriate for the species and planting site.

After optimizing the arrangement of the population, we wanted to evaluate that we were satisfied with the outcome. This is because the function *f* is complex, and we wanted to check that the minimization of this value also represented an improvement in terms of more intuitive measures. To do this, we compared the optimized arrangement to a random arrangement (the initial random arrangement, for simulated annealing) in terms of the spatial distances between plants exceeding selected thresholds of genetic similarity. Next, we recognized that one random arrangement might be unrepresentative. We therefore generated a set of 1000 random arrangements, and for each of these, calculated the number of pairs of genetically similar plants that were close together.

## 3 RESULTS

### 3.1 Empirical data sets: summary of in situ population genetics

The raw SNP data sets for *P. spicata* and *Eucalyptus* sp. Cattai each consisted of tens of thousands of markers, of which 6076 and 2509 (respectively) had complete data and were polymorphic in the condemned populations that were examined in detail. General features of these data sets are summarized in Table 1.

**TABLE 1:** A summary of population genomic data sets for *Pimelea spicata* and *Eucalyptus* sp. Cattai. For each species, we describe the aggregate properties of the genomic data set, including the number of samples, populations and SNP markers (before and after filters were applied), and the range of values is provided for allelic richness and *F*_IS_. This study is concerned with one condemned population of each species, and summary statistics for these populations are provided. For *P. spicata*, we provide separate estimates for Airport site samples that were collected as part of this study, and samples that were collected previously and propagated at the Australian Botanic Garden, Mt Annan. For *E*. sp. Cattai, we provide estimates for adult trees (excluding seedlings), unless otherwise indicated

	*Pimelea spicata*	*Eucalyptus* sp. Cattai
Species		
Samples	282 (270^a^)	188^b^ (152^a^)
Populations	16	8
SNP loci		
Raw	41,334	68,355
Preliminary filters	22,449	14,288
Allelic richness^c^	1.25–1.81	1.55–1.69^d^
*F*_IS_ ^c^	−0.09 to 0.23	−0.07 to 0.25^d^
		
Condemned population	Airport (Propagated)	Saltwater
SNP loci [>2%]	6076 [3087^e^]	2509 [1057^e^]
Samples	100 (16^a^, ^f^)	18^a^ adult, 59^a^, ^g^ seedling
Allelic richness	1.81 (1.78^f^)	1.69 adult, 1.68 seedling
*F*_IS_	0.22 (0.13^f^)	0.18 adult, 0.34 seedling

^a^ After excluding samples on the basis of clonality or high levels of missing data.

^b^ A total of 308 samples of *Eucalyptus* species were sequenced together and will be used for taxonomic delineation in a future study.

^c^ Range of values among sampled locations.

^d^ Adult trees only.

^e^ The number of SNPs that were common in the condemned population.

^f^ Samples collected previously from airport site and propagated at the Australian Botanic Gardens, Mt Annan.

^g^ After excluding four putative interspecific hybrids from ex situ collection designs.

For *P. spicata*, we set the threshold for identifying highly genetically similar individuals to kinship = 0.4 (see Appendix S1). Based on this criterion, we identified seven sets of highly similar individuals. These possibly represented seven genets each with multiple ramets, or groups of closely related, inbred individuals. For each group of highly similar samples, we identified the sample missing the fewest SNP genotypes and excluded the remainder from the subsequent analyses. In sum, 12 samples were removed from the data set due to very high levels of genetic similarity. *Pimelea spicata* populations all had small, positive, *F*_IS_ values, except one population that had *F*_IS_ = −0.09. These positive values of *F*_IS_ indicate there was an excess of homozygosity relative to the level expected based on allele frequencies. The condemned airport population had a high level of allelic richness, and a large value of *F*_IS_, relative to the other populations (Table 1).

In *E*. sp. Cattai, two samples were removed due to very high levels of missing genotype data (>50%). We used a threshold of kinship = 0.45 to identify highly similar individuals of *E*. sp. Cattai and remove them from the data set (see Appendix S1). On this basis, we removed a total of 34 samples, including eight adult trees from the condemned Saltwater population. *Eucalyptus* sp. Cattai populations all had *F*_IS_ ranging from small negative values (minimum = −0.07) to small positive values (maximum = 0.25). The condemned Saltwater site had the greatest observed levels of allelic richness, with 1.69 for adult trees, and 1.68 for seedlings.

### 3.2 Allele capture in propagation populations

**FIGURE 3:**
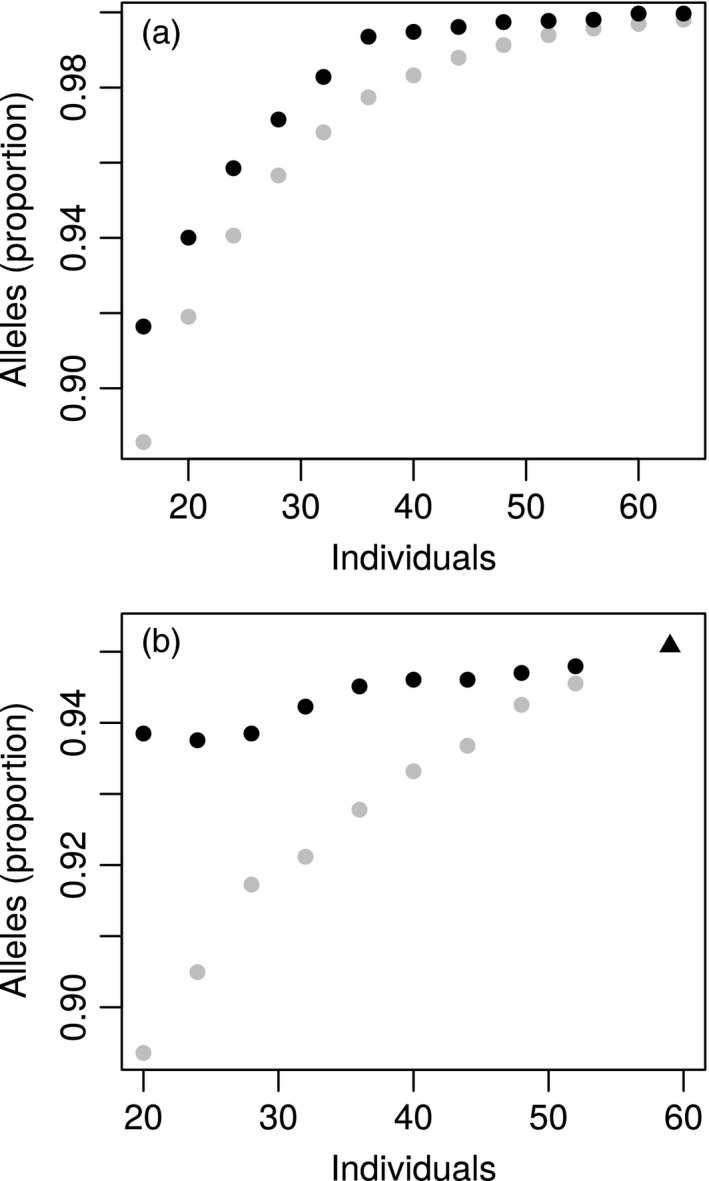
The proportion of loci that was polymorphic in different candidate propagation populations of (a) *Pimelea spicata* and (b) *Eucalyptus* sp. Cattai. Different numbers of individuals (horizontal axis) were selected from the condemned populations (airport and saltwater, respectively) by maximizing gene diversity (black symbols) and by choosing individuals at random (grey symbols, means of 100 replicates). For each of these populations, we calculated the proportion of SNP loci that were polymorphic (vertical axis). These analyses consider loci where the minor allele was common (allele frequency >2% and >3%, in *P. spicata* and *E*. sp. Cattai, respectively) in the condemned population. The triangular point for *E*. sp. Cattai represents the use of all available seedlings

We designed propagation populations using individuals from condemned populations of *P. spicata* and *Eucalyptus* sp. Cattai. We examined the proportion of common SNPs in the condemned population that were polymorphic in the propagation populations (i.e. biallelic SNPs where both alleles were ‘captured’ in the propagation population). For both species, the proportion of SNPs that were captured increased as a function of the size of the propagation population (Figure 3). These relationships were decelerating, such that beyond a certain size of propagation population, adding more individuals had a small effect. In *E*. sp. Cattai, the maximum proportion of SNPs that was captured was smaller (95.1%; Figure 3b), because some alleles that were common in the adult tree population were absent from the seedlings that were available for the propagation population. The propagation populations whose composition was optimized (for maximum gene diversity) tended to capture more SNPs than those whose composition was random. However, this effect was greatest for small propagation populations and smaller in larger propagation populations. The magnitude of this pattern also differed slightly between the species. In *E*. sp. Cattai, the mean number of SNPs captured in a small (*N*_P_ = 20) random population was 95.2% of the number of SNPs captured in an optimized population of equal size (Figure 3b), while in *P. spicata*, the corresponding value was 97.7% (Figure 3a).

**FIGURE 4:**
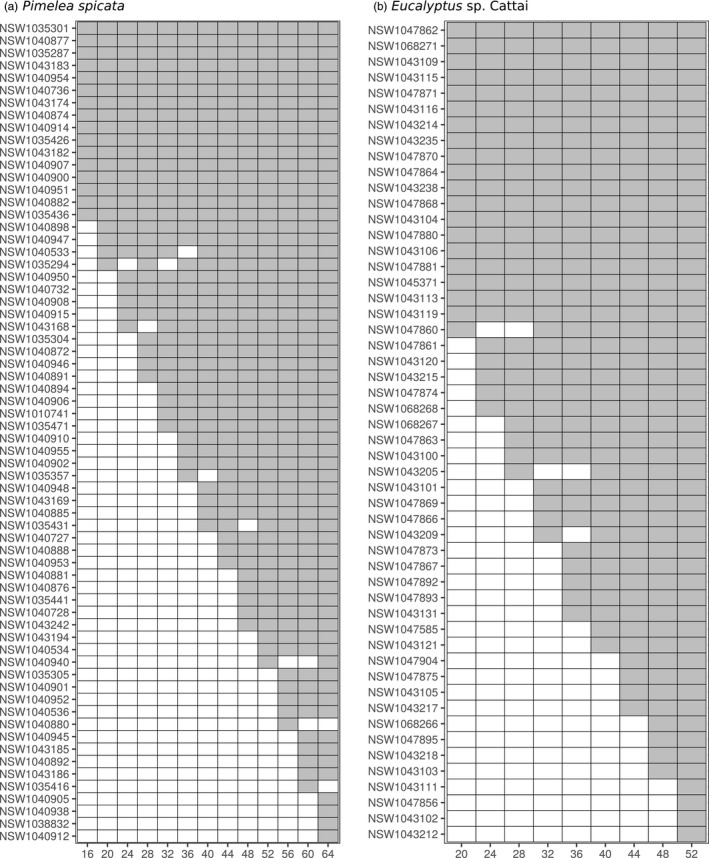
The individual plants included in propagation populations of different sizes for (a) *Pimelea spicata* and (b) *Eucalyptus* sp. Cattai. Filled (grey) cells in the matrix indicate the plants that were included in an optimized population of a nominated size (horizontal axis). Individual plants are listed by unique identifiers (starting ‘NSW…’) that were allocated to the corresponding leaf tissue samples. The individuals are arranged in order of their first appearance in a translocation population. Individuals that were not included in any of the translocation population designs (*N* = 50 for *P. spicata*, and *N* = 7 for *E*. sp. Cattai) are omitted from these displays. For each species nestedness is apparent, whereby plants that were included in small optimized populations tend also to be included in larger optimized populations

For both species, the optimized propagation populations of different sizes were highly ‘nested’ in terms of the individual plants that were included (Figure 4). That is, individuals that were included in the smallest propagation populations were almost always included in larger populations.

### 3.3 Allele loss in translocation populations

**FIGURE 5:**
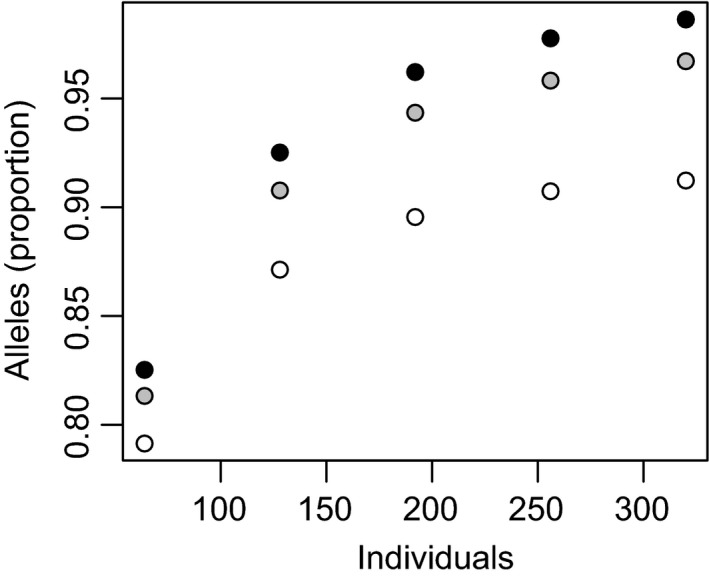
Simulations of genetic polymorphism for translocation populations of different sizes for *Pimelea spicata*. Potential translocation populations of different sizes (*N*_T_: 64, 128, 192, 256, 320 individuals) were assembled using propagation populations of different sizes (*N*_P_: 16, 32 and 64 individuals). For example, to construct a translocation population of 192 individuals from a propagation population of 32 individuals, 6 ramets (192/32 = 6) of each individual in the propagation population are used. Each translocation population was simulated forwards for 10 generations. At the conclusion, the number of SNP loci that remained polymorphic was calculated and expressed as a proportion of the total number of loci where the minor allele was common (>2%) at the airport. These proportions are plotted as a function of the size of the translocation population, for propagation populations of 16 (white circles), 32 (grey circles) and 64 (black circles) individuals (respectively)

For *P. spicata*, we assembled candidate translocation populations of different sizes from optimized propagation populations and performed forward simulations to examine their performance in conserving genetic diversity (Figure 5). In general, large translocation populations (many plants) lost fewer alleles over the course of 10 generations than smaller translocation populations (fewer plants). However, the increase in the proportion of alleles that were maintained as a function of translocation population size was decelerating, such that the benefit of increasing the size of the translocation population beyond 196 individual plants was relatively modest. The size of the propagation population that was used to assemble the translocation populations also had a substantial effect on the proportion of alleles that were conserved in the translocation population. When translocation populations were assembled using cuttings from small propagation populations (i.e. when few genets were used), the number of alleles conserved after 10 generations appeared to be limited by the proportion that were represented in the propagation population.

### 3.4 The trade‐off between genetic diversity and using local material

**FIGURE 6:**
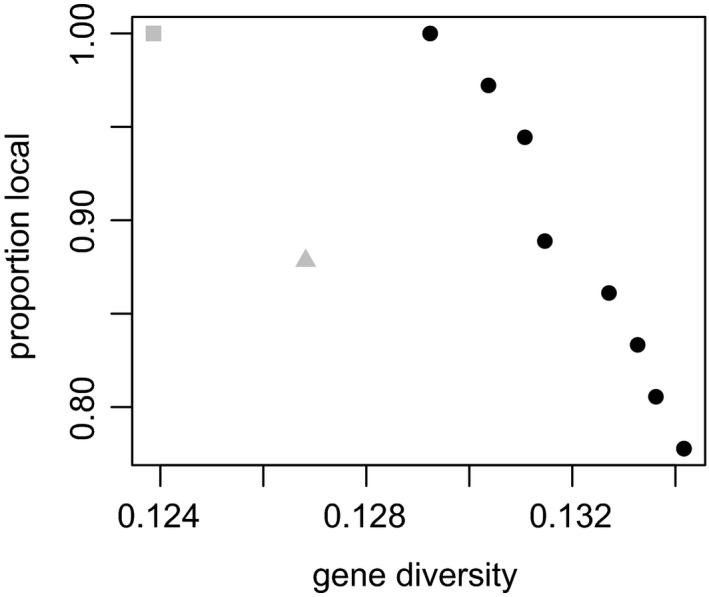
The gene diversity (horizontal axis) of optimized collections (*N*_P_ = 36) of *Eucalyptus* sp. Cattai seedlings from a condemned population. Multiobjective optimization was used to identify populations that maximized diversity when different proportions of individuals from a single source population were used (vertical axis). For optimized populations, there was a negative association between the gene diversity of collections and the use of local material (circles). That is, using individuals from additional populations tended to promote genetic diversity. We also selected populations at random that did (grey triangle) and did not (grey square) incorporate nonlocal seedlings (points represent means of *n* = 100 randomly selected collections)

In *Eucalyptus* sp. Cattai, we wanted to understand how incorporating plants from other sites in addition to the condemned population influenced the genetic diversity of the ex situ collection. Multiobjective optimization characterized a trade‐off between the gene diversity of a collection (*N*_P_ = 36 plants), and the proportion of individuals from the local (condemned) population (Figure 6). That is, populations with greater proportions of nonlocally sourced seedlings (and smaller proportions of locally sourced seedlings) tended to have greater levels of gene diversity than populations that consisted exclusively of seedlings from the condemned population. Populations whose composition was optimized, even without individuals from different populations, tended to have greater gene diversity than randomly selected populations (Figure 6).

### 3.5 Optimizing the spatial arrangement of a *Eucalyptus* sp. Cattai planting

**FIGURE 7:**
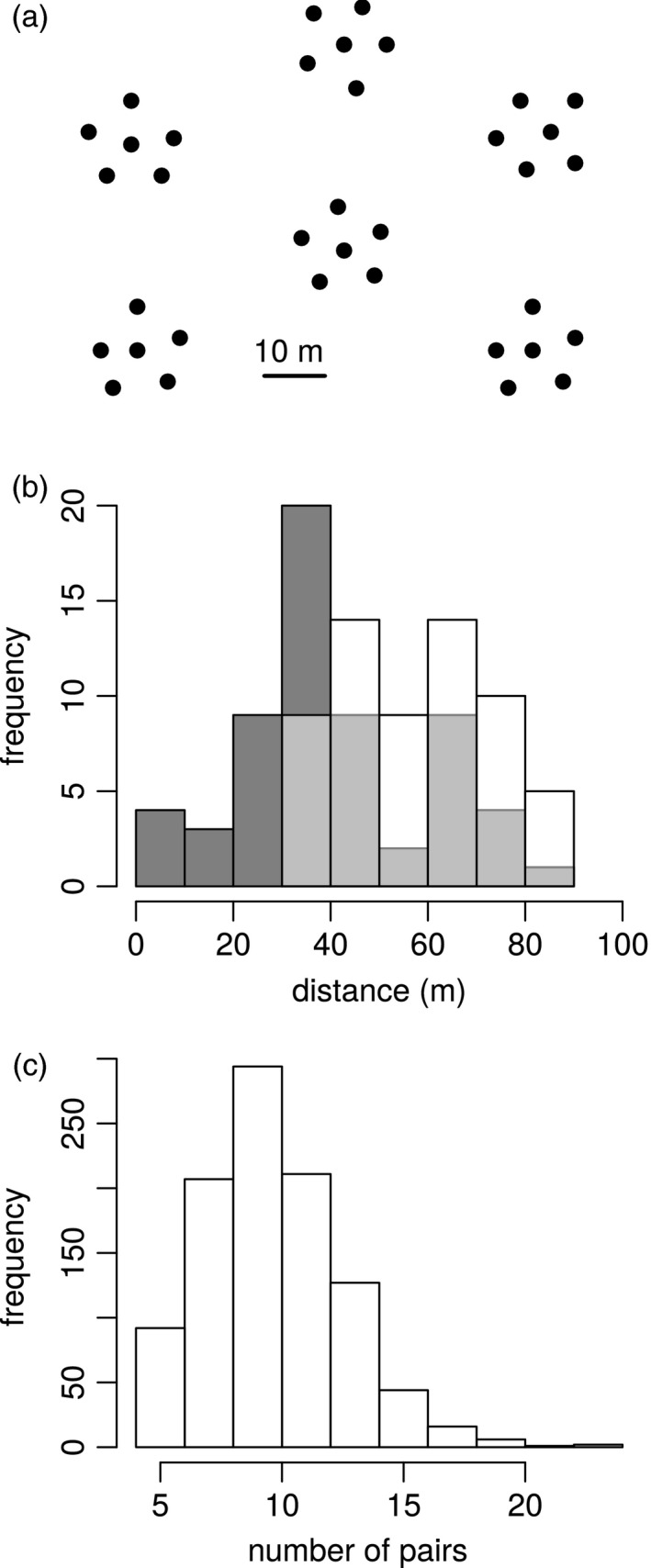
Optimization of the spatial arrangement of individuals from a condemned population of *Eucalyptus* sp. Cattai. The arrangement of individuals of *E*. sp. Cattai on a nominal spatial layout (a) was optimized using a simulated annealing algorithm. For the optimized arrangement and a single random arrangement, we show (b) the frequency distributions of pairwise spatial distances between plants with kinship ≥0.12 (with white and dark grey columns, respectively, and light grey where these overlap). The optimized population had fewer pairs of genetically similar plants close together, and more that were far apart. We calculated the number of pairs of genetically similar plants (kinship ≥0.12) that were close together (spatial distance ≤25 m) in each of 1000 randomly arranged populations of the same individuals (c). All have a greater value than the optimized arrangement, which was zero

We arranged a population of 36 *Eucalyptus* sp. Cattai individuals to minimize a value, *f*, which describes the spatial proximity of genetically similar individuals. At the start of the simulated annealing chain, when the samples were arranged randomly, *f* was 0.00069. At the end of the chain, an arrangement had been found where *f* was 0.00037. However, the function *f* is complex, so we wanted to check that this solution represented an improvement according to more intuitive measures. We observed that the optimized solution had no cases where genetically similar plants (kinship ≥0.12) were close together (spatial distance ≤25 m), whereas the (initial) random solution had nine such cases. Also, the optimized solution had 38 cases where genetically similar plants were spaced far apart (distance >50 m) while the (initial) random arrangement had 16 (Figure 7b). Further, out of 1000 random arrangements, the mean number of pairs of genetically similar plants (kinship ≥0.12) that were close together (spatial distance ≤25 m) was 10.1 pairs (Figure 7c), compared to zero pairs in the optimized arrangement.

## 4 DISCUSSION

### 4.1 A broadly applicable workflow for preserving genetic diversity

This study examined two plant species that have become endangered due to habitat destruction. They are representatives of a much larger group of imperilled plant species, globally, whose ranges overlap areas that have been subject to heavy and continuing disturbance (IUCN, 2020). Where the destruction of a population is unavoidable (a population is ‘condemned’), it may be possible to preserve its genetic diversity in a new environment by translocation. We set out to explore a broadly applicable workflow (Figure 1) for doing this efficiently, in terms of the number of plants that need to be maintained. The implementation of different parts of the workflow must vary according to the properties of particular species or conservation objectives. To illustrate, we applied the workflow to two exemplar species with substantially different life‐history and horticultural properties (Figure 1) – *P. spicata* can be readily grown from cuttings, while *Eucalyptus* sp. Cattai must be propagated from seeds. Parts of the workflow were very similar for the two species, such as the selection of material for an ex situ collection based on genotype data (Figure 1c,d). Other steps in the workflow needed to be approached quite differently, such as the amplification of material for translocation (Figure 1e,f). Below we discuss the steps in the workflow (Figure 1) in relation to these two exemplar species.

### 4.2 The design of propagation populations

For both species, we examined the establishment of a propagation population, consisting of a subset of individuals of the condemned source population. We used an optimization algorithm to select the individuals for this population in a way that maximized genetic diversity (Marshall & Brown, 1975) and that was similar to previous approaches (Diniz‐Filho et al., 2012; Richards et al., 2007). In both species, we observed that when the propagation population was small, the optimization of its composition led to a substantial improvement in the proportion of SNPs that was captured. Conversely, when the propagation population was quite large, most common alleles were represented in the average randomly chosen population, and the benefit of optimization was modest. These findings are broadly consistent with the observations of many previous empirical and simulation studies (e.g. Bataillon et al., 1996, Hoban et al., 2020, Hoban & Schlarbaum, 2014, Richards et al., 2007, Schoen & Brown, 1993).

Despite these similarities, two differences between our exemplar data sets merit consideration. The first was that in *P. spicata*, it was possible to design propagation populations that captured essentially all of the common alleles (>99%) that were observed in the condemned population. In contrast, in *E*. sp. Cattai, there was an upper limit on the proportion of alleles from the population of adult trees that was captured in a propagation population derived from seedlings. Fortunately, this upper limit was relatively large (around 95.1% of SNPs), but this serves as a useful reminder that the quantity and type (seeds vs. cuttings) of material that is collected initially places constraints on the preservation of genetic diversity of condemned populations (Griffith et al., 2015; Namoff et al., 2010). A second difference between *P. spicata* and *E*. sp. Cattai was the magnitude of the difference between small populations (e.g. *N*_P_ = 20) that were optimized and random. It seems likely that properties of different condemned populations, and the ways in which they must be sampled, will affect the diversity that can be gained by optimizing small collections. For instance, if the individuals that are available from a condemned population are clustered in family groups of highly unequal sizes, it might be especially useful to ‘balance’ the available diversity using genotype information. Similar observations have been made in relation to the benefit of balancing the representation of individuals from different groups ranging from different families at the local scale, to different ancestral population groups at the landscape scale (e.g. Diniz‐Filho et al., 2012, Richards et al., 2007, Schoen & Brown, 1993).

For condemned populations, we suggest that characterizing the diversity captured for different strategies, and comparing these for populations with different properties, will provide much insight about the circumstances where optimization of collection composition is especially useful.

### 4.3 Amplification strategies: design and refine

When we set out to preserve the genetic diversity in a condemned population, our ultimate goal is usually to establish a self‐sustaining population in a new location (Guerrant, 1996; Vallee et al., 2004). The propagated collection could be maintained during the establishment of one or more translocation populations for the purpose of supplementation following the loss of genotypes, or as a contingency in case of the catastrophic loss of a translocated population. However, usually we would not want to assume the cost of maintaining a collection on an indefinite basis, as might be the case for a germplasm core set for an important crop species. Therefore, we wanted to examine the maintenance of diversity in translocation populations that were assembled from different propagation populations. In general, we found that after 10 generations (a time horizon of 20–40 years for *P. spicata*) of simulated evolution, larger populations maintained a greater proportion of the diversity from the condemned population. This effect was largely saturated in translocation populations with more than 200 individuals. The size of the propagation population (number of genets in the collection) that was used to assemble the translocation population also had a substantial effect, essentially imposing an upper limit on the amount of diversity that was maintained. This meant the propagation population size was limiting to the diversity represented in large translocation populations. In sum, we concluded that if there was a premium on the efficient use of propagation resources, a reasonable strategy for *P. spicata* was to maintain an optimized propagation population of 32 plants and to use these in establishing a translocation population of 256 plants, or 8 ramets of each of the 32 selected genets. We predicted this strategy would preserve polymorphism at 95.8% of genetic loci identified in the source population. However, we note there is substantial uncertainty associated with this quantitative prediction, owing to the possibility of greater attrition of polymorphism during propagation (Fant et al., 2016; Hoban, 2019; Richards et al., 2010), or following establishment of a translocated population.

There can be substantial variation in propagation success among plants of the same species. In this sense, optimized designs for ex situ conservation represent a target that would maximize an objective, but in some cases cannot be achieved. Similarly, it is likely there will be variation among genets in survival rates in translocation populations following establishment. For these reasons, it might be useful to supplement a planned translocation population with alternate genets from the ex situ collection before or after establishment (Kashimshetty et al., 2012). Approaches are available that permit most members of a population to be ‘locked in’, while a search is conducted to identify plants that could be added to it for an optimal outcome (e.g. , Bragg et al., 2020, Gouesnard et al., 2001). In the context of this uncertainty concerning propagation success and survival, we were particularly interested to observe a substantial ‘nestedness’ in the way individual plants were incorporated into successively larger propagation plantings (Figure 4). That is, population designs of different sizes provided a potentially useful index for the diversity value of each plant, likely reflecting a combination of the heterozygosity of the plant, and its genetic distance to other available plants.

This indicator of the potential contribution to diversity of different plants could usefully guide decisions about the allocation of effort towards the propagation of different genets, or for working iteratively towards the best achievable outcome, following the death of established plants. However, we note that it is unclear whether this nestedness would be present for populations that were optimized for different objective criteria and might be stronger or weaker in different collections of plants.

### 4.4 Supplementing local diversity and ‘swamping’

In conservation programmes, it is sometimes necessary to decide whether to manage the preservation of a group of organisms as a unit (Moritz, 1994) or to deliberately incorporate individuals from conspecific populations to promote diversity and avoid inbreeding depression (Fitzpatrick et al., 2020). We wanted to quantify the trade‐offs involved in this kind of decision, balancing the advantage of supplementing the genetic diversity in the translocated population, while limiting the risk of ‘swamping’ local alleles. To do this, we used multiobjective optimization to design propagation populations with maximized gene diversity and that were supplemented with different numbers of conspecific seedlings from different populations. In general, adding individuals from different populations tended to increase the level of gene diversity, as would be expected on the basis that these individuals would be less redundant (Schoen & Brown, 1993) than the material from the same population. However, in this case, we also found that propagation populations with greater genetic diversity could be obtained by optimizing the composition of individuals from the condemned population, than by choosing individuals at random and supplementing with individuals from different populations. This highlights the potential value of genotyping and carefully managing the composition of the ex situ collection, relative to a sensible alternative. More broadly, we note that in other cases this outcome would depend heavily on factors such as the variation available within the condemned population and the level of genetic differentiation between the populations. The main point we want to stress is that it is possible to characterize the trade‐off between the maximization of diversity and the level of swamping among different candidate collections, so that a manager can make informed decisions based on the level of mixing they are willing to tolerate, and the amount of diversity that can be gained as a result. This is applicable to many decisions beyond the management of condemned populations, and especially in the management of species that are at risk from swamping by a closely related species, which is common in threatened eucalypts (e.g. Field et al., 2009; Rutherford et al., 2019).

### 4.5 The spatial arrangement of a conservation planting

For species such as *Eucalyptus* sp. Cattai, if we wanted to amplify the quantity of material that is available for a translocation population, we would need to establish a planting, and collect progeny. We examined a planting with ‘clumped’ locations, with the aim of promoting overall seed production (McCallum et al., 2019). However, we also wanted to promote the genetic diversity of the progeny, by keeping genetically similar individuals spatially separated. We found that a simple optimization algorithm was successful at separating similar individuals in space. Similar algorithms have been used extensively in designing different kinds of conservation actions with spatial components (e.g. Ball et al., 2009). We note that the utility of this approach, and the way in which it is best implemented, would depend heavily on properties of the species and the population in which it is implemented (Kashimshetty et al., 2012). Optimization of spatial arrangement could be beneficial when pollinators tend to travel short distances between visits to flowers, but is likely to be negligible when pollinators travel large distances. There is also substantial scope to modify the optimization in light of information about pollination biology. In particular, the function we minimize, *f*, describes a ‘penalty’ associated with the distance and genetic similarity of each pair of individuals. This function could be changed, for example, to prioritize the separation of individuals with genetic similarity exceeding *x* by a spatial distance exceeding *y*.

## 5 CONCLUSIONS

Here we studied populations of endangered plant species that will be lost due to planned habitat destruction. For each of these two ‘condemned’ populations, we implemented a workflow to preserve the existing genetic diversity effectively and efficiently in an optimized ex situ collection and examined strategies for establishing diverse translocation populations using these collections. Consistent with previous observations, we were able to capture a large proportion of the genetic polymorphism represented in each source population with a reasonably modest number of plants (>32), provided these plants were carefully chosen. We found that 256 vegetative cuttings of *P. spicata* from such a collection could preserve a substantial proportion of genetic variation (>95% of polymorphism) over an appropriate time frame. We also described strategies for designing a planting of *Eucalyptus* sp. Cattai to promote the production of genetically diverse seed for translocation. These workflows are united by a common framework (Figure 1), which can be applied in diverse ways to accommodate species with different life‐history and horticultural properties.

## CONFLICT OF INTEREST

The authors declare there are no conflicts of interest.

## Supporting information

Appendix S1

## Data Availability

Genetic data for *P. spicata* and *Eucalyptus* sp. Cattai are available in Dryad (repository doi:10.5061/dryad.5hqbzkh53). Some analyses performed for this study and described in detail in Appendix S1, used code from online repositories available here: https://github.com/jasongbragg/OptGenMix, https://github.com/jasongbragg/Ramet, https://github.com/jasongbragg/PlantPopGenFit. Scripts used for implementing analyses performed for *P. spicata* and *Eucalyptus* sp. Cattai are available in Dryad (repository doi:10.5061/dryad.5hqbzkh53).
