# Supplementary material for: Conserving the genetic diversity of condemned populations: Optimizing collections and translocation
Source: Evol Appl. 2021 Feb 1;14(5):1225–38. doi: 10.1111/eva.13192 (PMC8127699; doi:10.1111/eva.13192)
Supplement: Supplementary file 1 — Appendix S1 [file EVA-14-1225-s001.docx]

**Supporting Information: “Conserving the genetic diversity of condemned populations:**

**optimizing collections and translocation”**

**S1. Optimization: details of implementation**

*Overview*

For each species, we had a set of genotyped plant samples, and wanted to choose a subset of the plants (of size *N_S_*) for an *ex-situ* collection in a way that maximized the gene diversity of the collection. The simulated annealing (Kirkpatrick et al. 1983) optimization algorithm is a good fit for this problem, in that it examines different possible discrete solutions (*e.g.*, different subsets of a set of plants, of a given size) and searches for one that approximately optimizes an “objective function” (*e.g.*, the maximization of gene diversity). The specific methods we used here to apply simulated annealing to the optimization of a plant population for conservation purposes has been described previously (Bragg et al. 2020), and therefore we provide a brief overview, with a focus on details that are most pertinent to the present study.

Simulated annealing begins with a solution that is chosen at random. In our case, this involves choosing the subset of plants (size *N_S_*) at random. It then proceeds to refine the solution, in relation to the nominated objective function, over the course of a number of steps (a ‘chain’ of steps). At each step, a small change to the ‘current’ solution is generated at random, and proposed. In our case, the proposed solution is generated by randomly dropping one individual from the current solution (*i.e.*, the current subset of chosen plants), and randomly replacing it with another. If the gene diversity of the proposed subset of plants is greater than the current subset, the proposal is accepted, and the proposed subset becomes the current subset, for the next step in the chain. If the gene diversity of the proposed subset of plants is smaller than the current subset, the proposal still may be accepted, but with a probability that declines as a function of the magnitude of the difference between the two values of gene diversity (*δ*), and that also declines over the course of the chain. Specifically, the probability is given by *e*^(-^*^δ/T^*^)^, where *T* is a variable that declines over the course of the chain from a parameterized maximum value, and is called the ‘temperature’ of the chain as an analogy to the process of annealing in metallurgy. This means that proposals that are poorer in terms of the objective function, or that have lower levels of gene diversity, are more likely to be accepted if the difference in levels of gene diversity is very small, or early in the chain. This aims to give the algorithm opportunities to traverse many different solutions early in the chain, and to escape ‘local optima’, before converging on an (approximately) optimal solution. Simulated annealing was implemented with approaches presented by Bragg et al. 2020, and using an implementation (in R; R Core Team, 2016) of the algorithm that is available here: https://github.com/jasongbragg/OptGenMix. The code we used to call this algorithm for each species is available in the dryad repository associated with this article (doi:10.5061/dryad.5hqbzkh53).

*Genetic diversity*

When choosing populations with maximized gene diversity, we ran simulated annealing chains of 10,000 steps. We ran several preliminary chains and inspected the outputs before choosing a maximum temperature for each species. Our goal was to find a value for the maximum temperature parameter where gene diversity meandered slightly as a function of chain length at the beginning of the chain, before rising to a maximum value. For *Pimelea spicata*, we used a value of 0.00005, and for *Eucaltpyus* sp. Cattai, we used a value of 0.0001.

*Spatial arrangement*

We also used simulated annealing to optimize the spatial arrangement of a collection of 36 *Eucalyptus* sp. Cattai individuals across a ‘clumped’ grid of locations (see Fig 7A). The goal was to keep genetically very similar individuals far apart spatially, to reduce the probability that they would pollinate each other, and in turn, to promote genetic diversity in the progeny from the population. To characterize the degree to which closely related plants were close together, we first calculated, for each pair of plants *i* and *j*, the value of their genetic similarity (kinship), *G_i,j_*, multiplied by the reciprocal of their distance apart in space across the grid, *S_i,j_* (in arbitrary units), or: (1 / *S_i,j_*) × *G_i,j_*. This value is large for pairs of plants that are genetically very similar and very close together. We estimated the mean of this value across all pairs of plants, and used this as the objective function in a simulated annealing chain. Specifically, we chose an arrangement of plants that approximately minimized this value, using a simulated annealing chain of 50,000 steps, and a maximum temperature of 0.00001. The chain began with the individuals arranged randomly on the grid, and at each step a proposed arrangement was generated by randomly choosing two plants, and swapping their locations. The algorithm we used to optimize the spatial arrangement of the individuals is available here: <https://github.com/jasongbragg/OptGenMix>, and the code we used to call this algorithm for *Eucalyptus* sp. Cattai is available in the dryad repository associated with this article (doi:10.5061/dryad.5hqbzkh53).

*Supplementing diversity*

We also recognized that in some cases, individuals from a single (condemned) site might exhibit relatively low levels of genetic diversity, and that it might be beneficial to supplement this diversity by incorporating individuals from other sites (*e.g.*, to reduce the risk of inbreeding depression). However, if the central goal is to preserve the variation that exists at the condemned site, the incorporation of individuals from other populations might be undesirable, if the genetic contribution from other sites (and that remains intact in the ‘wild’) ‘swamps’ the variation derived from the condemned site. Therefore we wanted to have an approach that would allow us to characterize the possible tradeoff between using a larger proportion of individuals from different sites, and obtaining increased levels of genetic diversity as a result. We did this using a multiobjective simulated annealing algorithm, very similar to one presented in Bragg *et al.* (2020). Briefly, the multiobjective algorithm was similar to simulated annealing with a single objective, except at each step, we ask whether proposed subsets of individuals were better than the current subset for both objective criteria (*i.e.*, a larger proportion of individuals from the condemned site, and larger gene diversity). If the proposed subset was poorer for values of one or both of these measures, the proposal was accepted with a probability that decreased as a function of the progression through the chain. During the chain, the multiobjective simulated annealing algorithm collects a ‘nondominated archive’ of solutions, where one solution ‘dominates’ another if it is better for values of both objectives. This means a nondominated archive traces the optimal tradeoff curve between the two variables (termed the ‘Pareto front’). For the multiobjective optimization presented here, we first performed an optimization to find optimal solutions at two extremes – a solution maximizing gene diversity using only individuals from the condemned population, and a solution maximizing gene diversity using any number of the available individuals from other populations. We then started multiobjective optimizations from both these starting points (parameters: steps = 20,000, max temperature = 2, minimum temperature = 0.001), and combined the nondominated archives. Finally, to ensure we had found an optimized tradeoff curve, we began an additional multiobjective optimization starting from each point on the initial nondominated archive. Here we used lower values for temperature parameters, to focus the search on improved solutions (parameters: steps= 10,000, max temperature = 0.01, c1 = 0.05, c2 = 0.05, cboth=1, minimum temperature = 0.00005). The very simple multiobjective simulated annealing algorithm we used is available here: https://github.com/jasongbragg/OptGenMix. The code we used to call this algorithm for *Eucalyptus* sp. Cattai available in the dryad repository associated with this article (doi:10.5061/dryad.5hqbzkh53).

**S2. Simulations**

For *Pimelea spicata*, we wanted to evaluate the potential performance of different alternative translocation populations by running forward simulations. To do this, we used a simple population genetic forward simulator (the code is available here: https://github.com/jasongbragg/PlantPopGenFit). We started each simulation with a candidate translocation population, and iterated forward for 10 generations. We chose 10 generations because it corresponds to about 20-50 years for *Pimelea spicata*, which seemed like a reasonable time horizon for setting management goals. The simulation had non-overlapping generations, meaning that each generation, each individual died and was replaced by a new recruit. Each recruit was generated by selecting a mother and father plant from the previous generation. At each locus, the recruit inherited one allele at random from each of the mother and father. The parents of each recruit were selected at random. Specifically, the simulator generates a phenotype value for each individual from the genotype data (genotype effects on the phenotypes are prescribed), and then uses a fitness function to determine the probability, for each potential recruit, that each individual in the population was the mother and father of the recruit, respectively. Here, we had little informational about the mating system or likely levels of variation in reproductive success in *Pimelea spicata*. We therefore parameterized the simulations so that each individual had essentially an equal probability of being a parent to each recruit. We allowed selfing to occur (*i.e*, the same plant could be mother and father to a recruit), parameterized so that for a selected mother plant, the probability of self-fertilization was equal to the probability of fertilization by each other plant in the population. We did this with the expectation that selfing can occur, though based on moderate observed *F_IS_* values (Table 1), it does not seem to occur extensively. The code we used to perform the simulations is available in the dryad repository associated with this article (doi:10.5061/dryad.5hqbzkh53).

**S3. Setting thresholds for genetic similarity**

For each species, we wanted to identify highly genetically similar plants for removal, so there were not ‘duplicate’ plants in the datasets, and especially those that were used for designing collections. To do this, we characterized the genetic similarity between pairs of individuals using a measure of kinship, as described in the main text. We estimated kinship among individuals within each population, and not between members of different populations. This is because this approach uses estimates of population allele frequencies, and if these differ between populations, inferred values of kinship between individuals might be affected. We also calculated the Euclidian distance between the genotypes (scored at each locus as the number of copies of an arbitrary reference allele) of each pair of individuals (using the R dist() function). We visualized the relationship between these measures for each species to help guide decisions (Fig S1.1). These analyses were implemented using code that is available here: <https://github.com/jasongbragg/Ramet>, as well as scripts that are available in the dryad repository associated with this article (doi:10.5061/dryad.5hqbzkh53).

For *Pimelea spicata*, there appeared to be a discrete group of pairs of samples with higher levels of genetic similarity than the remainder of the pairs (large values of kinship, small distances, shown in blue in Fig S1.1a). We then found clusters of highly similar individuals. These were possibly the ramets of different genets, though it was also possible these represented closely related, inbred individuals. To do this, we found all pairs of individuals with a value of kinship exceeding a threshold (0.4). We then created a network (using R package igraph, Csardi and Nepusz, 2006) in which pairs of individuals that exceeded this threshold shared an ‘edge.’ We identified clusters as the ‘components’ of this network (using the components() function of igraph). We found this useful because it imposed transitivity in the assignments of individuals to several of the clusters – that is, if pair A and B exceeded the threshold, and B and C exceeded the threshold, but pair A and C did not, it placed A, B, and C together in a cluster (and visual inspect of Fig. S3.1 suggests this was a reasonable outcome).

For *Eucalyptus* sp. Cattai, it was more difficult to identify a discrete group of pairs with substantially smaller values for kinship, and we selected a threshold of 0.45. We note that no seedlings from the Saltwater population (the focal individuals for conservation purposes) were removed based on this criterion.

| 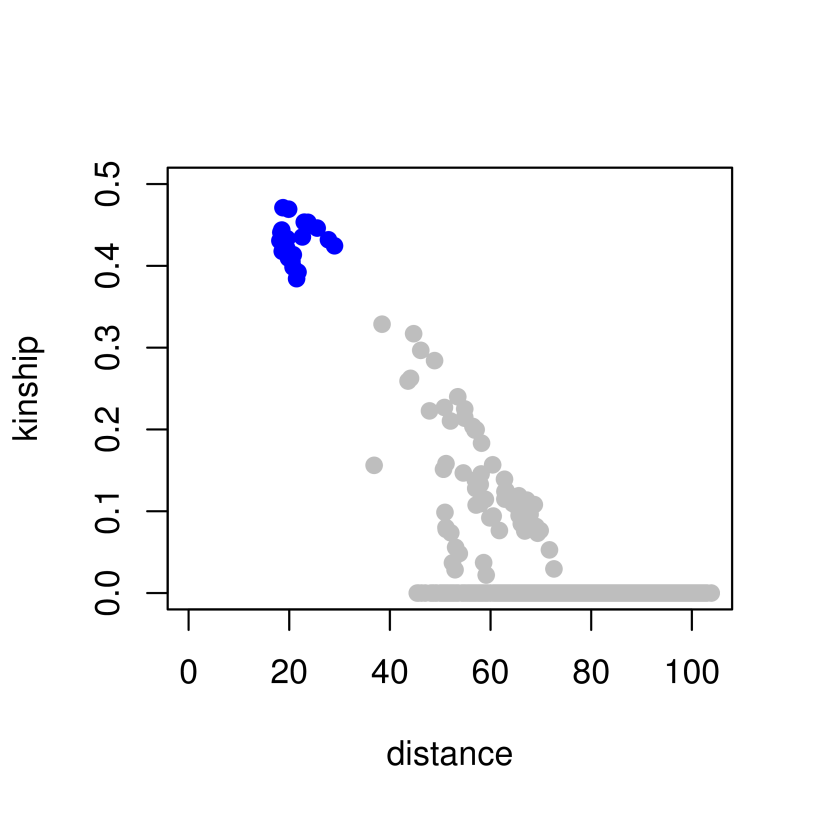  A |
| --- |
| 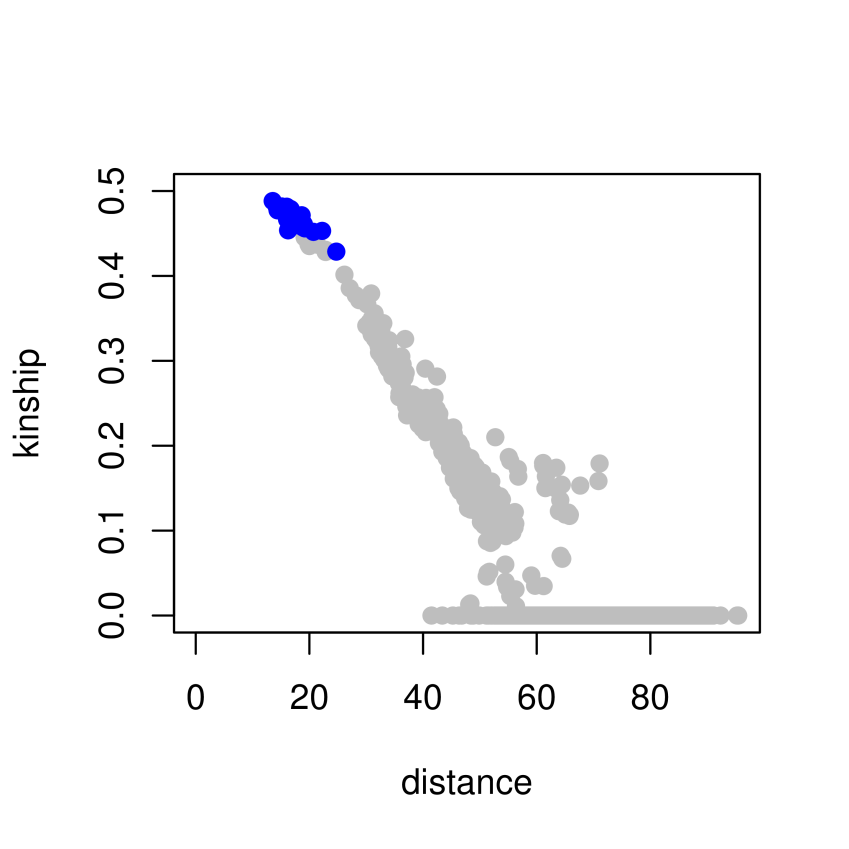  B |
| **Fig S3.1.** Genetic similarity thresholds that were used for (A) *Pimelea spicata* and (B) *Eucalyptus* sp. Cattai. For pairs of samples, kinship is plotted as a function of genetic distance. In each case, a kinship threshold (0.4 and 0.45, respectively) was imposed, and ‘clusters’ of highly similar individuals were identified according to that threshold. Pairs of individuals belonging to these clusters are shown in blue, others in gray. Note, for *E.* sp. Cattai, points are only shown for pairs of individuals within the same population. This is because adults and seedlings were analyzed separately downstream, and therefore kinship analyses were applied within these populations. For *Pimelea spicata*, we also calculated kinship within populations, but here it seemed reasonable to assume that pairs of individuals from different individuals had kinship=0, and these values have been displayed in the plot. |

References

Csardi G., Nepusz T. (2006). The igraph software package for complex network research. *InterJournal, Complex Systems*, 1695.

R Core Team (2016) R: A language and environment for statistical computing. R Foundation for Statistical Computing, Vienna, Austria.
